# Investigation of eight candidate genes on chromosome 1p36 for autosomal dominant total congenital cataract

**Published:** 2008-09-30

**Authors:** Kathryn P. Burdon, Kathryn Hattersley, Salil A. Lachke, Kate J. Laurie, Richard L. Maas, David A. Mackey, Jamie E. Craig

**Affiliations:** 1 Department of Ophthalmology, Flinders University, Adelaide, Australia; 2 Division of Genetics, Brigham and Women's Hospital, Harvard Medical School Boston, MA; 3 Centre for Eye Research Australia, University of Melbourne, Royal Victorian Eye and Ear Hospital, Melbourne, Australia

## Abstract

**Purpose:**

To identify the causative gene for autosomal dominant total congenital cataract in a six-generation Australian family displaying linkage to chromosome 1p36.

**Methods:**

Eight candidate genes (*HSPB7*, *FBXO42*, *EFHD2*, *ZBTB17*, *CAPZB*, *FBLIM1*, *ALDH4A1*, and *MFAP2*) from within the previously defined linkage interval were selected based on expression in lens and their known or putative function. The coding exons were sequenced in multiple affected family members and compared to the reference sequence.

**Results:**

No segregating mutations were identified in any of the eight genes. Thirty-one polymorphisms were detected, 20 of which were in the exons and 11 in the flanking introns.

**Conclusions:**

Coding mutations in *HSPB7*, *FBXO42*, *EFHD2*, *ZBTB17*, *CAPZB*, *FBLIM1*, *ALDH4A1*, and *MFAP2* do not account for congenital cataract in this family.

## Introduction

Congenital cataract is a relatively rare condition leading to severe visual impairment or blindness in affected children. The disorder is highly heterogeneous with 17 genes reported to date and an additional 16 loci reported for which the gene remains to be identified. The known genes to date include structural proteins (lens crystallins and cytoskeletal proteins), transport molecules (gap junctions and aquaporins), and transcription factors (developmental and stress response). All forms of Mendelian inheritance (autosomal dominant and recessive as well as X-linked) have been reported.

One region of interest in congenital cataract is the short arm of chromosome 1, particularly 1p36. At least two independent loci in three families have been mapped to this region [1-3]. In 1995, Eiberg and colleagues [1] mapped the Danish Volkmann-type cataract (OMIM 115665) to a region between the 1p telomere and marker D1S214. Shortly after this in 1997, Ionides and colleagues [2] mapped posterior polar cataract in a three-generation British family to a similar but larger region between the telomere and D1S2845. More recently, complete congenital cataract displaying autosomal dominant inheritance in a six-generation Australian family was localized to a six megabase (Mb) region between markers D1S228 and D1S199, which contains 60 annotated genes [3]. This region overlaps with that of the British family but not the Danish family. Thus, there are at least two congenital cataract loci on 1p36.

**Figure 1:**
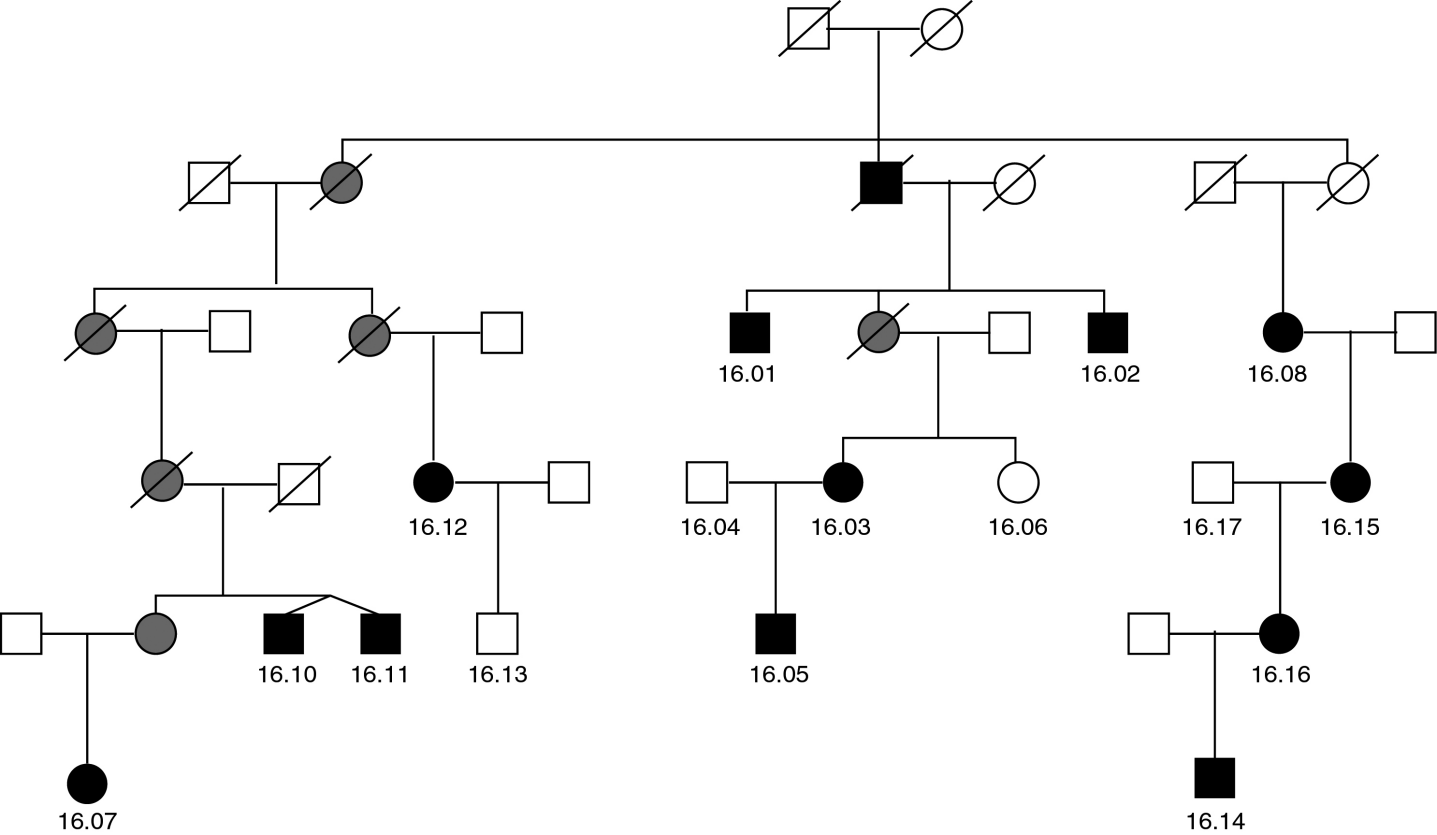
Six-generation Australian pedigree with total congenital cataract mapped to chromosome 1p36. Family members who provided samples and were examined by an ophthalmologist are indicated by an identification number. Unexamined obligate carriers are shaded gray.

This report describes the investigation of eight positional and functional candidate genes for complete congenital cataract in the Australian family previously described (Figure 1) [3]. The genes were chosen on the basis of lens expression and their known or putative function and were confined within the known linkage interval in our family. They have a variety of functions including stress response, ubiquitination, calcium binding, cytoskeleton association, and metabolism (Table 1).

**Table 1:** Known/putative function and ocular expression of candidate genes.

**Gene**	**Known/putative function**	**Expression in human lens (NEIbanklibrary)**	**Expression in other ocular tissue (NEIbanklibrary)**	**Other comments**
*HSPB7*	Small heat shock protein, related to alpha-crystallins		NbLib0065 (fetal eye)	Alpha-crystallins associated with congenital cataract
*FBXO42*	Ligation of ubiquitin to proteins to signal as targets for degradation	NbLib0019, NbLib0021	NbLib0065 (fetal eye), NbLib0079 (whole eye), NbLib0042 (retina), NbLib0072 (rpe/choroid)	Ubiquitin pathway implicated in lens fiber development and age-related cataract
*EFHD2*	Calcium binding protein	NbLib0019, NbLib0021	NbLib0065 (fetal eye), NbLib0124 (fetal eye)	Calcium regulates calpains, which cleave crystallins
*ZBTB17*	Transcription factor, responds to cytoskeleton and cell-cell adhesion	NbLib0068		Cytoskeleton is important in maintaining lens integrity
*CAPZB*	Regulates growth of actin filament in cytoskeleton	NbLib0019, NbLib0021 NbLib0068,		
*FBLP1*	Cytoskeletal protein, localizes to cell-cell and cell-ECM junctions			
*ALDH4A1*	Proline metabolism	NbLib0068	NbLib0065 (fetal eye), NbLib0124 (fetal eye)	Excess proline may accumulate in tissues
*MFAP2*	Component of elastin microfibrils		NbLib0065 (fetal eye), NbLib0124 (fetal eye)	High expression in mouse embryonic lens

The identifiers of NEIbank EST libraries in which each gene is reported are given. rpe=retinal pigment epithelium.

HSPB7 (heat shock 27 kDa protein 7) is a small heat shock protein that is involved in cellular response to stress. The α-crystallins are members of the small heat shock protein family and are well known to cause autosomal dominant and recessive forms of congenital cataract [4-7]. FBXO42 (F-box only protein 42) is an E3 ubiquitin ligase, which is involved in the ligation of ubiquitin to target proteins to signal for protein degradation via the proteasome. During lens fiber development, the cells lose their organelles through targeted ubiquitination, and an incomplete degradation of these organelles can result in cataract [8]. The pathway has also been implicated in age-related cataract [9]. EFHD2 (EF hand domain family member D2) is likely a calcium-binding protein. It is expressed in many tissues including lens and fetal eye. Excess calcium in the lens has been shown to cause congenital cataract in rabbits [10], mice [11], and sheep [12]. Calcium activates a family of intracellular proteases known as calpains, some of which are lens-specific [13]. Calpains cleave crystallins causing them to precipitate and aggregate [14].

The cytoskeleton is known to be important in congenital cataract with several mutations reported in the lens specific cytoskeleton protein genes, *BFSP1* and *BFSP2* [15-17]. Several genes in the linkage region are associated with the cytoskeleton. ZBTB17 (zinc-finger and BTB domain-containing protein 17, also known as myc-interacting protein 1) is a transcription factor that interacts closely with microtubules [18] and activates gene transcription in response to changes in the cytoskeleton and to cell-cell adhesion formation [18,19]. CAPZB is a subunit of CAPZ, an actin-binding protein that regulates growth of the actin filament by capping the barbed end of the growing filament [20]. FBLIM1 (filamin-binding LIM protein 1, also known as migfilin) is a cytoskeleton protein that localizes to cell-cell adhesions, cell-ECM (extracellular matrix) junctions, and the nucleus, where it may also play an important role in transcriptional activation [21]. Links between the actin cytoskeleton and the ECM are important in maintaining tissue integrity and play a part in controlling cell morphology and behavior [21].

ALDH4A1 (aldehyde dehydrogenase 4A1) is a mitochondrial matrix NAD(+)-dependent dehydrogenase, which catalyzes the second step of the proline degradation pathway, converting pyrroline-5-carboxylate to glutamate. Mutations in this gene lead to the recessive disorder hyperprolinemia type II (OMIM 239510), which is characterized by a build up of both proline and pyrroline-5-carboxylate in the blood. The accumulation of excess materials in the lens is seen in hereditary hyperferritinemia cataract syndrome (OMIM 600886) and galactosemia (OMIM 230400), where elevated levels of ferritin (an iron storage protein) and galactose respectively lead to cataract. *MFAP2* was chosen because of high expression levels in the mouse lenses from embryonic days 10.5-12.5 (data not shown). MFAP2 (microfibril-associated protein 2) is a protein component of elastin microfibrils, which are structural components of the extracellular matrix. Although there is no known direct link between the extracellular matrix and congenital cataract, a defect in the structure of microfibrils may affect the transparency of lens during development.

## Methods

Approval for the study was given by the Clinical Research Ethics Committees of the Royal Victorian Eye and Ear Hospital (Melbourne, Australia) and Flinders University (Adelaide, Australia). The family and linkage region on 1p36 has been previously described [3]. A six-generation family with total congenital cataract was ascertained (Figure 1), and DNA was extracted from whole blood or buccal swabs. Linkage analysis at known congenital cataract loci was conducted, and significant linkage was detected on chromosome 1 between markers D1S228 and D1S199. *PAX7* (involved in ocular development) was previously excluded as a cause of cataract in this family. DNA from all family members was subjected to whole genome amplification using GenomiPhi (GE Healthcare, Rydalmere, NSW, Australia) according to the manufacturer’s protocols to increase the stock of DNA for sequencing candidate genes. Amplified samples were diluted 1:20 for polymerase chain reaction (PCR). Any variants detected in this DNA were confirmed in genomic DNA.

Candidate genes from within the linkage region were chosen on the basis of expression in the human lens or fetal eye either from evidence in publicly available databases including UniGene and NEIbank or from the lens microarray database generated by Lachke and Maas (unpublished). Putative or known functions were also considered. Primers (Appendix 1) were designed to amplify the coding exons (including splice sites) of each gene, and PCR products were directly sequenced on the ABI PRISM 3100 Genetic Analyzer (Applied Biosystems, Foster City, CA) with BigDye Terminators (Applied Biosystems) according to standard protocols. Each exon was sequenced in two affected individuals, and sequences were compared to the reference sequence. Due to limited amounts of DNA, different individuals were sequenced for each gene. In some cases, an unaffected descendant was also investigated. Common previously reported variants were not considered a high priority for follow-up in the additional affected or unaffected individuals. A segregating polymorphism with limited information available in public databases (rs41310410) was genotyped in 77 unrelated individuals using the SNaPshot® multiplex kit (Applied Biosystems) to determine its frequency. A primer directly adjacent to the polymorphic nucleotide was annealed to the PCR product, and a single fluorescently tagged ddNTP was incorporated according to the manufacturer’s protocols (Applied Biosystems). The products were then electrophoresed on the ABI PRISM 3100 Genetic Analyzer to detect the alleles. The potential of this synonymous single nucleotide polymorphism (SNP) to affect splicing was investigated in silico using the ESEfinder [22,23]

## Results and Discussion

**Figure 2:**
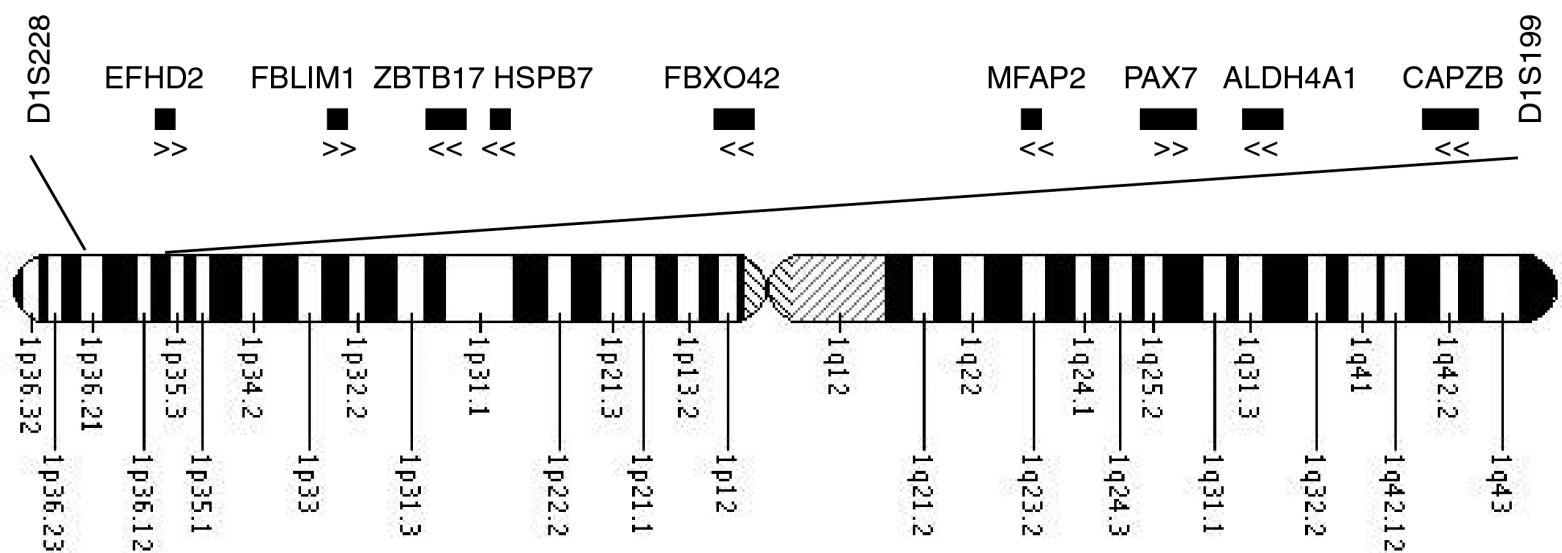
Ideogram of chromosome 1 showing approximate location of the linkage region and each candidate gene. The flanking microsatellite markers are shown. The direction of transcription of each gene is indicated with arrows below the symbol. Note that gene size and position are approximate only. Sixty genes are found in this region in total.

Annotated genes with a function or putative function that could be linked to cataract formation and a known expression in human lens or fetal eye were chosen for sequencing. The fetal eye was considered to be an appropriate tissue due to the congenital (and thus developmental) nature of the phenotype in this family. The genes chosen were *HSPB7*, *FBXO42*, *EFHD2*, *ZBTB17*, *CAPZB*, *FBLIM1*, *ALDH4A1*, and *MFAP2* (Appendix 1, Figure 2). *PAX7* was previously excluded by direct sequencing [3].

Protein coding exons and the intron-exon boundaries of all eight genes were directly sequenced. Multiple polymorphisms were identified in the coding exons and surrounding introns of these candidate genes (Table 2). No segregating mutations were identified. Of the 31 polymorphisms detected, 20 were in exons.  Four of the exon polymorphisms were non-synonymous. All variants were represented in the dbSNP database. Common or intronic SNPs were not investigated further. Frequency information and validation were not available for several reported polymorphisms including the non-synonymous SNP rs35196193 in *FBXO42* and the synonymous rs41310410 in *ALDH4A1*. Based on the individuals sequenced, rs35196193 was excluded due to lack of segregation while segregation of rs41310410 could not be excluded. The minor allele of rs41310410 may introduce a binding site for proteins involved in splicing of the mRNA, specifically the SRp40 protein [22,23]. Such sites are known as exonic splice enhancers and can improve (or may be necessary for) the efficiency with which introns are removed from the mRNA. Thus, this SNP was assessed in all family members for segregation. All affected individuals carried the A allele, and three of these (16.08, 16.10, and 16.11) were homozygous for this allele, suggesting it to be common in the population. To confirm it as a common polymorphism, it was genotyped in 77 unrelated, unaffected individuals, revealing that the A allele occurs at a frequency of 3.2% in the Australian population. One case of homozygosity was detected. This indicates that this SNP is found in the normal population and thus is not likely to be the cause of severe congenital cataract in this family.

**Table 2:** Polymorphisms identified in candidate genes in family with total congenital cataract.

**Gene**	**No. coding exons**	**Affected samples sequenced**	dbSNP **identifier**	**Location of variant**	**Codon**	**MAF freq in**dbSNP	**Reference allele**	**Genotype affected 1**	**Genotype affected 2**	**Genotype Unaffected 16.13**
*HSPB7*	3	16.07, 16.15	rs1572381	Exon 1	5′ UTR	0.30*	C	TC	CC	TT
			rs945416	Exon 1	S19S	0.38	C	TC	CC	TT
			rs732286	Exon 1	A33A	0.44	T	CT	TT	TT
			rs1739840	Exon 3	T117T	0.35	T	TC	TT	CC
*FBXO42*	9	16.08, 16.15	rs2273311	Exon 2	S5S	0.21*	G	AG	AA	
			rs12069239	**Exon 10**	**A471P**	**0.36**	G	CG	CC	
			rs35196193	**Exon 10**	**T509A**	**N/A**	G	GA	GG	
*EFHD2*	4	16.08, 16.15								
*ZBTB17*	14	16.08, 16.15	rs848217	Exon 6	A207A	0.11	T	CT	CT	CT
			rs9661939	Exon 8	F334F	0.15**	C	CC	CC	CT
			rs12134932	Intron 8		N/A	G	GT	GT	GG
*CAPZB*	9	16.08, 16.15	rs169957	Intron 5		0.37	C	CA	CA	
*FBLIM1*	7	16.08, 16.15	rs12146078	Intron 5		0.21	C	TT	CT	
			rs10927851	**Exon 6**	**F191S**	**0.24**	C	TT	TT	
*ALDH4A1*	15	16.02, 16.05	rs941495	Intron 3		N/A	A	GG	GG	GG
			rs6426611	Intron 7		0.10#	C	CC	CC	CT
			rs28497538	Intron 9		N/A	A	CA	CA	AA
			rs6426813	Intron 9		0.41	G	CT	TT	TT
			rs2230705	Exon 10	A350A	0.23	C	CC	CC	CG
			rs41310410	Exon 10	P362P	0.03###	G	GA	GA	GG
			rs2230706	Exon 12	A407A	0.33##	A	GA	GA	GG
			rs7550938	Exon 12	S140S	0.27	A	GA	GA	GA
			rs2230707	Exon 12	A417A	0.33	C	CT	CT	CC
			rs7550822	Intron 12		N/A	A	GG	GG	GG
			rs2230708	Exon 13	D460D	0.21	T	CT	CT	
			rs2230709	**Exon 13**	**I470V**	**0.14**	G	GA	GA	
			rs35657817	Intron 14		N/A	G	GA	GA	
			rs11740	Exon 15	3′ UTR	0.38	G	GA	N/A	AA
*MFAP2*	8	16.03, 16.08	rs2235932	Intron 3		0.33	C	TT	TT	
			rs761422	Exon 6	H144H	0.44	A	GG	GG	
			rs761423	Intron 6		0.49	T	CC	CC	
			rs1051225	Exon 9	3′ UTR	0.45	T	TT	CC	

The number of coding exons sequenced is given. Exons representing only the untranslated regions were not examined. Non-synonymous variants are highlighted in **bold**. The minor allele frequency (MAF) reported in dbSNP is shown. Where possible, this is the lowest reported value for Caucasian populations. If unavailable, the value for other ethnic groups is shown as indicated. An asterisk indicates an Asian sample; a double asterisk denotes an African American sample; a single hashmark (#) denotes an African sample; the double hashmark (##) indicates that it is a mixed sample; and three hashmarks (###) denotes allele frequency determined in this study in 77 unrelated controls. The reference allele and genotypes for family members sequenced are also given.

From these data, we conclude that none of these eight genes are involved in the development of this severe and highly penetrant congenital cataract, although regulatory mutations have not been ruled out. Of 56 annotated genes in this linkage region, nine (including *PAX7*) have now been excluded. At least 10 additional genes show lens or fetal eye expression and will form the next group of genes to be assessed in this family.

## Appendix 1. Primer sequences and expected product size for PCR and sequencing of coding exons.

To access the data, click or select the words “Appendix 1”. This will initiate the download of a (pdf) archive that contains the file. Where an intron was small enough, exons were combined into a single amplimer. Where an exon was too large, it was sequenced as overlapping fragments (denoted by lower case letters after the exon number).
